# Depth: a web server to compute depth, cavity sizes, detect potential small-molecule ligand-binding cavities and predict the pK_a_ of ionizable residues in proteins

**DOI:** 10.1093/nar/gkt503

**Published:** 2013-06-12

**Authors:** Kuan Pern Tan, Thanh Binh Nguyen, Siddharth Patel, Raghavan Varadarajan, M. S. Madhusudhan

**Affiliations:** ^1^Bioinformatics Institute, 30 Biopolis Street, #07-01, Matrix, Singapore 138671, ^2^School of Computer Engineering, Nanyang Technological University, Singapore 639798, ^3^Department of Biological Sciences, National University of Singapore, Singapore 117543, ^4^Molecular Biophysics Unit, Indian Institute of Science, Bangalore 560012, India and ^5^School of Biological Sciences, Nanyang Technological University, Singapore 637551

## Abstract

Residue depth accurately measures burial and parameterizes local protein environment. Depth is the distance of any atom/residue to the closest bulk water. We consider the non-bulk waters to occupy cavities, whose volumes are determined using a Voronoi procedure. Our estimation of cavity sizes is statistically superior to estimates made by CASTp and VOIDOO, and on par with McVol over a data set of 40 cavities. Our calculated cavity volumes correlated best with the experimentally determined destabilization of 34 mutants from five proteins. Some of the cavities identified are capable of binding small molecule ligands. In this study, we have enhanced our depth-based predictions of binding sites by including evolutionary information. We have demonstrated that on a database (LigASite) of ∼200 proteins, we perform on par with ConCavity and better than MetaPocket 2.0. Our predictions, while less sensitive, are more specific and precise. Finally, we use depth (and other features) to predict pK_a_s of GLU, ASP, LYS and HIS residues. Our results produce an average error of just <1 pH unit over 60 predictions. Our simple empirical method is statistically on par with two and superior to three other methods while inferior to only one. The DEPTH server (http://mspc.bii.a-star.edu.sg/depth/) is an ideal tool for rapid yet accurate structural analyses of protein structures.

## INTRODUCTION

Atom/residue depth measures the degree of burial of an atom/residue from bulk solvent (1). This simple measure has found a variety of uses in characterizing physical and chemical properties of protein structures. It has been shown to correlate well with hydrogen/deuterium amide proton exchange rates (1,2), structural stability (1), sizes of globular domains (1,3), hydrophobicity (1,3,4), residue conservation (4), protein activity and 3D structural model accuracy (5). Further, residue depth has been used to predict the location of folding nucleation sites (4,6), protein–protein interaction hot spots (1), phosphorylation sites (4) and small molecule-binding sites on proteins (7).

This study reintroduces our web server to compute residue depth. Here, we have explored three applications of residue depth, namely, estimating the sizes of cavities in proteins, improving protein ligand-binding site prediction and predicting acid dissociation constant (pK_a_) for ionizable amino acids GLU, ASP, LYS and HIS.

Our algorithm, DEPTH, inherently differentiates between bulk solvent waters and waters present in protein cavities. These cavities could be of structural and/or functional importance. For instance, cavities in the interior could destabilize proteins, whereas some cavities on the exterior could bind ligands. Here, we describe the depth-based algorithms to compute the volumes of these cavities and predict ligand-binding sites.

pK_a_ is a measure of the protonation strength of ionizable groups. Properties of proteins such as folding, stability, solubility, dynamics, interactions and functions in general could all be modulated by pK_a_ (8–14). pK_a_s of ionizable amino acid residues are sensitive to their immediate protein/solvent environment. As depth is a concise way of describing the residue environment in proteins, we have used it here in conjunction with other features such as accessible surface area (ASA), electrostatic interactions and hydrogen bonds to predict pK_a_.

Amino acid protonation (or deprotonation) is sometimes not accurately described by a single value. Our method, like many others, however simplifies the problem and predicts one value of pKa that is most representative of the interaction between the ionizable group and its immediate environment. Accordingly, we have benchmarked our method against pK_a_ values that have been experimentally determined unambiguously.

In the sections later in the text, we describe our methods and show the benchmarks of our predictions. For each of the applications of depth described later in the text, we have compared our method with other popular methods and tested the statistical significance of the differences in results. Finally, we briefly describe the functioning of our web server.

## MATERIALS AND METHODS

Residue (or atomic) depth measures the closest distance of the residue (or atom) to bulk solvent. We have described the computation of this feature in detail earlier (1,7). In the sections later in the text, we outline methods to compute the sizes of cavities and detect which of these are likely to bind small molecule ligands and compute the pK_a_ of ionizable residues of proteins.

### Detection of cavities in proteins

The depth of protein residues are computed by distinguishing between bulk and non-bulk waters. Briefly, the protein of interest is solvated [immersed in a box of SPC216 waters (15)] a number of times by varying its orientation [for a detailed description of the method, see (1,7)]. Water molecules with less than a certain number of neighbours (in this study—less than 2 waters within 4.2 Å) are deemed non-bulk. Residue depth is computed as the average distance to the closest bulk solvent molecule from each solvation iteration.

All non-bulk water molecules are considered to be contained in cavities. The solvated protein structures from the different iterations are superimposed using CLICK (16,17). Waters from different iterations are clustered together if they lie within 1.2 Å of each other. A cavity is identified if it contains at least two water molecules. Each cavity contains a set of sometimes overlapping water molecules that however do not clash with protein atoms. The volumes of these water-containing cavities are measured using a Voronoi procedure, a modification of a protocol described earlier (18). Volumes are computed for the protein with all water molecules and then again for the same system without the non-bulk waters using the program McCavity (19). The difference in these two measurements gives us the initial estimate of cavity volume. McCavity on average slightly overestimates volume (Supplementary Table SA1), and hence the results were re-calibrated using a linear fit: V_c_ = m_1_V + m_2_. Where V_c_ is the expected volume, V is the volume computed by McCavity. The values of constants m_1_ and m_2_ (0.8 and 21, respectively) are obtained from a least squares fit of calculated to expected volumes. The output to our program distinguishes between cavities that are buried and exposed. All cavities that are lined by residues whose minimum depth is greater than 3.75 Å are considered buried cavities, or inaccessible to bulk solvent.

#### Datasets

The relatively larger residues VAL, LEU, ILE, MET, PHE and TRP were all mutated *in silico* to ALA in 40 different positions in 13 proteins (Supplementary Table SA1). The mutations were effected by simply deleting all side chains atoms after the CB atom. No minimization was performed. The volumes of the cavities thus created are expected to be the differences in molecular volume between the large amino acid and ALA (19). This data set was split randomly into training and testing sets of 20 mutants each. The volume calibration described earlier in the text used the training set data only. Another data set of 34 proteins was compiled from the PDB (Supplementary Table SA2). These proteins were cavity-containing point mutants of RNase S, Barnase, Gene V protein, T4 Lysozyme and Human Lysozyme. Experimentally determined changes to protein stability, in terms of free energy change (ΔΔG^o^ values), are available for all 34 mutants.

### Small molecule ligand-binding site prediction

Previously (7), we developed a simple method to predict small molecule ligand-binding sites based on the observation that ligand-binding residues on proteins were simultaneously deep and accessible to water. The accuracy of our method was comparable with that of other more sophisticated methods such as LIGSITE (20), Pocket-Finder (21) and SURFNET (22). In this study, we have enhanced our prediction schema to include evolutionary information (in terms of residue conservation). The enhanced procedure consists of four consecutive steps.

(i) Assigning ligand-binding probability to residues. For every residue in a protein, its ligand-binding probability *P*_*i*_ was assigned based on its amino acid type *R*, depth *D* and solvent accessibility *S* from a database of 900 single chain ligand-bound proteins as
(1)
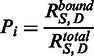

  A detailed description of this has been given earlier (7).
(ii) Adjustment to binding probability using evolutionary information. To incorporate evolutionary information, *P_i_* was adjusted with a conservation score *J_i_* as a weighted average to give the adjusted binding probability *q_i_*.
(2)
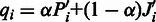

  where α is an optimized weighting coefficient (see end of this section for note on weight optimization), *P_i_*′ and *J_i_*′ are the normalized binding probabilities of residue and conservation score, respectively.
  The conservation score *J_i_* of a position *i* was defined as its Jensen–Shannon divergence with respect to a background distribution of amino acid residue occurrence. To compute Jensen–Shannon divergence, a multiple sequence alignment of homologues for the protein was obtained by running five iterations of PSI-BLAST (23) against the uniref90 sequence database (24) with an *e*-value cut-off of 0.0001. The Jensen–Shannon divergence *J_i_* at a position *i* is given by
(3)
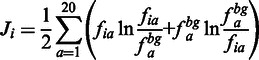

  where *f_ia_* is the frequency of residue a at position *i* and 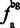 is the frequency of the residue a in the background distribution.
  In addition, pseudo-counts were introduced (25) to account for sparseness of data using the following formulae–
(4.1)
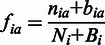

(4.2.1)
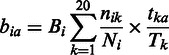

(4.2.2)
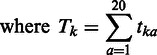

(4.3)
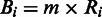

  where for amino acid a at position *i*, *f_ia_* is its occurring probability, n_ia_ is its frequency and *b_ia_* is its pseudo count. *N_i_* and *B_i_* are the total number of residue counts and pseudo counts at position *i*, respectively. *t_ka_* is the probability that amino acid *k* would be substituted by amino acid a as estimated from the BLOSUM62 matrix (26). *T_k_* is the overall probability of substituting amino acid *k*. *m* is a parameter that has been set to 5 and *R_i_* is the number of different residue types at position *i*.
  As conservation score *J* and binding probability *P* differ in magnitude, both measures were normalized to unity using
(5)
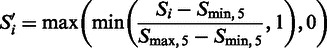

  where *S′_i_* is the normalized measure, *S*_min,5_ and *S*_max,5_ are the mean values of the smallest and largest five values of the respective measures.
(iii) Predicting cavity waters to be displaced. At every solvation cycle, using the adjusted residue-binding probabilities, we estimate for every cavity water (identified as described previously in section on detecting cavities), its likelihood to be displaced by a small molecule ligand. The displacement likelihood *D* is given by
(6)
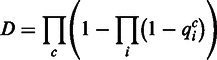

  where 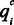 is the adjusted binding probabilities of residue i from chain c that is within 5.6 Å of the cavity water. We assume that displacement likelihood of a cavity water from two different chains are independent of one another [Equation (6)].
  Additionally, we made the assumption that a ligand must displace at least two water molecules (distance between displaced waters should not exceed 4.2 Å, i.e. 1.5 hydration shells) for a binding event to occur. Hence, for every cavity water, its neighbouring cavity waters within 4.2 Å are identified. The displacement likelihood of a cavity water was assigned as the average of the two highest displacement likelihoods of its and its neighbouring waters.
(iv) Prediction of binding site residues. A cavity water was predicted to be displaced when the averaged displacement likelihood exceeds a threshold value β. All residues with at least one atom within 6.5 Å of this cavity water were listed as binding site residues candidates. The candidate residues listed from different solvation iterations could be slightly different owing to differences in cavity water configurations. A residue was predicted to be part of the binding site if it was listed in >60% of the solvation iterations.
  The values of weighting coefficient α [Equation (2)] and threshold value β were optimized for the Matthews Correlation coefficient (MCC) (see ‘Results’ section) over a training set of 99 ligand bound PDB structures (Supplementary Table SB1), using a grid search of step 0.05 and 0.1, respectively. The optimized values of α and β were 0.7 and 0.8, respectively.

### Protein ionizable amino acid pK_a_ prediction

The model pK_a_ value of an ionizable amino acid residue (in this study, ASP, GLU, LYS or HIS) is determined by titrating isolated amino acids in solution (27). In the context of proteins, pK_a_ values are dependent on their immediate environments and could shift from these model pK_a_ values. We predict these shifts by characterizing the environment of ionizable groups using depth and other features.

The features used to describe the environment include (i) average depth of main-chain atoms (*DEPTH^MC^*), (ii) average depth of polar side-chain atoms (*DEPTH^polar SC^*), (iii) number of hydrogen bonds involving the ionizable groups as donor or acceptor (*HB*), (iv) The electrostatic energy, calculated in vacuum, of the interaction between the ionizable groups and their environments (truncated at a cut-off distance of 12 Å) (*EE*) and (v) percentage side-chain solvent ASA (*ASA^SC^*). For simplicity, these features are combined in the form of a linear combination
(7)
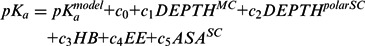

where c_0_-c_5_ are the coefficients of the linear combination.

Hydrogen bonds were detected between donor-acceptor pairs if they were (i) within 3.5 Å of one another and (ii) the donor-acceptor-acceptor antecedent angle was 100° or greater [adapted and modified from (28)]. For computation of electrostatics energy, hydrogen atoms were explicitly added using the program Reduce (29). All acidic groups were assumed deprotonated, whereas the basic groups were assumed protonated (HIS was assumed protonated at δ and ε positions). Partial charges were assigned to all atoms using values from the gromos43a1 force field (30). ASA was computed using the Shrake–Rupley algorithm (31).

#### Data sets and parameter optimization

The coefficients of the linear combination were obtained by optimizing the predictions on a training set of 112 ASP, 125 GLU, 70 LYS and 60 HIS residues, whose pK_a_ values were experimentally determined (Supplementary Table SC1). The coefficients of the linear combination for each of the amino acids were optimized separately. The prediction formula was then tested on a set of 15 GLU, 15 ASP, 15 LYS and 15 HIS (Supplementary Tables SC1 and SC3). None of the testing set data overlapped with the training set.

In the cases where the pK_a_s were determined for mutants of proteins, homology models were built using the mutate_residue command of MODELLER (32). In other cases where structures reported more than one alternative conformation for residues, the first listed conformation was always chosen.

The features of the linear combination here were selected from amongst a large number of features that were tested to describe residue environment (Supplementary Table SC2). Polar side chain atom depth and main chain atom depth were the most informative of the environmental features.

## RESULTS

### Cavity size estimation

We first tested the efficacy of our method to accurately compute the volumes of cavities in proteins. For this purpose, we chose the 20, *in silico*, large-to-small amino acid mutations that constituted the testing set (see Materials and Methods’ section). On average, we overpredict cavity volumes by about 0.9 Å^3^ (Table 1). We compared the performance of our method with those of McVol (33), VOIDOO (34) and CASTp (35). McVol and VOIDOO were run locally with default parameters while CASTp results were obtained by submitting the input PDB files to the server http://sts-fw.bioengr.uic.edu/CASTp/calculation.php. The errors in our method are consistently lower than those of the other methods compared (Table 1). Although McVol (average error: 1.3 Å^3^) performs statistically on par with our method, we are statistically significantly better than CASTp (average error: 23.2 Å^3^) and VOIDOO (average error: −96.1 Å^3^) according to a Wilcoxon paired sign rank test.

**Table 1.** Cavity size estimations by DEPTH, CASTp, VOIDOO and McVol

	DEPTH	CASTp	McVol	VOIDOO
Average error in cavity size estimation	0.9 Å^3^	23.2 Å^3^	1.3 Å^3^	−96.1 Å^3^
*P*-value		0.0002	0.4317	<0.0001

The *P*-values reported are from a Wilcoxon paired sign rank test applied to compare DEPTH with the other methods.

### Correlation with mutational stability

**Figure 1.**
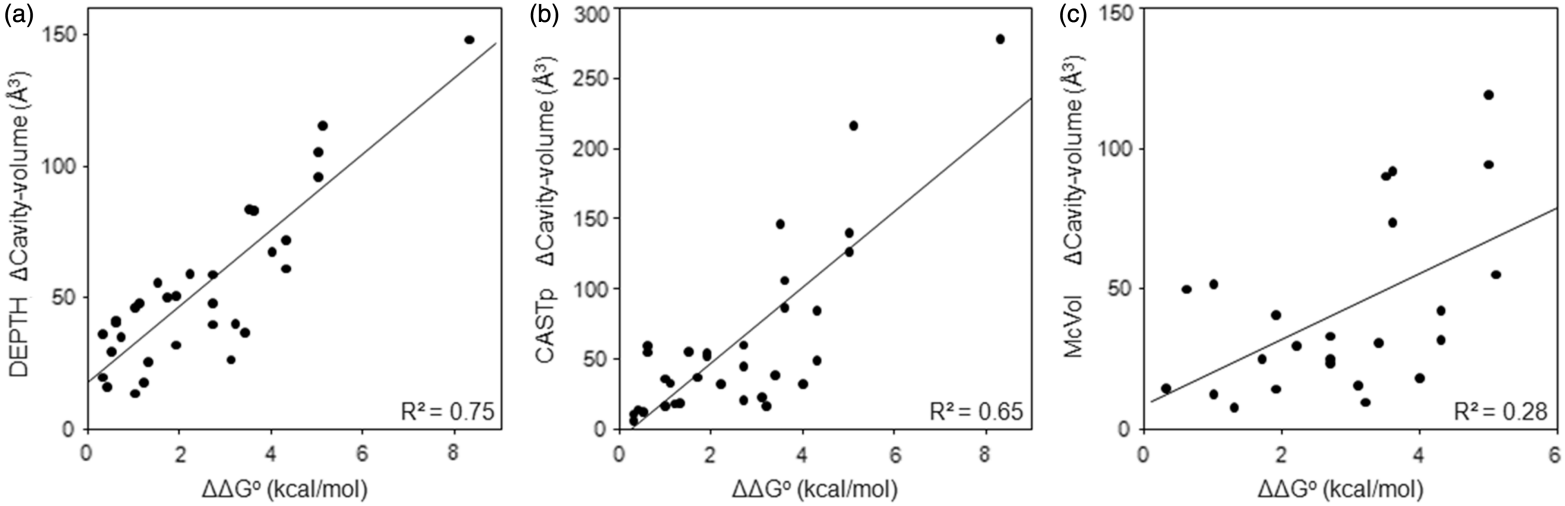
Correlation between experimentally measured free energy change on mutation (ΔΔG^o^) and cavity sizes computed by DEPTH (**a**), CASTp (**b**) and McVol (**c**).

We next tested how well our method, and the others, estimated the instability of mutations. For this, we used the data set of 34 crystal structures of single point (large-to-small) cavity containing mutants of RNAse S, Barnase, Gene V protein, T4 Lysozyme and human Lysozyme. Our volume estimates are better correlated (*r*^2 ^= 0.75, as compared with 0.65 and 0.28 for CASTp and McVol, respectively) to the experimentally determined free energy change (ΔΔG°) for each of the mutants (Figure 1). McVol, the next best method failed to predict cavities in 6 of the 34 cases while we detected all cavities except 1.

### Protein small molecule ligand-binding site prediction

#### Benchmark

In all, 299 proteins complexed to small molecule ligands were taken from the LigASite database v7.0 (36) for benchmarking. The data set consisted of 119 single- and 180 multi-chain proteins, filtered for non-redundancy (25%) and for which evolutionary information was available. This data set was divided into a training set of 99 structures (39 single-chain, 60 multi-chain) and a testing set of 200 structures (80 single-chain, 120 multi-chain). The training and testing sets do not overlap with each other (Supplementary Table SB1).

MCC (37) was computed to assess the performance of binary classification of residues (binding site or non-binding site) for each protein structure. It is computed as
(8)
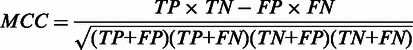

where TP, TN, FP and FN represent the rates of true positives, true negatives, false positives and false negatives, respectively. The overall performance of a predictor was measured as its mean MCC over the testing set.

We have compared our performance with the popular small molecule ligand-binding site predictor, ConCavity (38) and a meta-algorithm MetaPocket 2.0 (39). ConCavity adds evolutionary information to enhance three other popular binding site prediction methods—LIGSITE (20), Pocket-Finder (21) and SURFNET (22). The addition of evolutionary information makes ConCavity outperform the original methods (7,38). In our tests, ConCavity was run using LIGSITE for structural geometry with all parameters set to their default values (7,38). Evolutionary information for ConCavity was obtained at http://compbio.cs.princeton.edu/ConCavity/pqs/jsd/. MetaPocket is a consensus method that takes into consideration output from eight different binding site predictors including LIGSITEcs (40), PASS (41), Q-SiteFinder (42), SURFNET (22), Fpocket (43), GHECOM (44), ConCavity (38) and POCASA (45). MetaPocket 2.0 was run through the web-server at http://projects.biotec.tu-dresden.de/MetaPocket/, and the number of predicted binding sites was set to 1. MetaPocket 2.0 returns predictions for only a fraction of the LigASite testing set. The comparisons of MetaPocket to DEPTH and ConCavity were done on 110 proteins (70 single- and 40 multi-chain).

For single chain proteins, DEPTH performs on par with MetaPocket 2.0 (MCC: 0.55) and better than ConCavity (MCC: 0.53). For multi-chain proteins, DEPTH and ConCavity performed better (DEPTH MCC: 0.47, ConCavity MCC: 0.50) than MetaPocket 2.0 (MetaPocket 2.0 MCC: 0.33). The overall performance of DEPTH and ConCavity are similar (DEPTH MCC: 0.50, ConCavity MCC: 0.51), and a two-tailed paired *t*-test showed that the difference is not statistically significant (*P* = ∼0.8) The same test showed that both methods are significantly better than MetaPocket 2.0 (MetaPocket 2.0 MCC: 0.47) (Table 2).

**Table 2.** The MCC values for DEPTH, ConCavity and MetaPocket 2.0 binding site residue predictions over the testing set

	DEPTH	ConCavity	MetaPocket 2.0
Single-chain			
*N*	80	80	70
MCC	0.55	0.53	0.55
Difference		0.02	0.00
*P*-value		0.39	0.47
Multi-chain			
*N*	120	120	40
MCC	0.47	0.50	0.33
Difference		−0.02	0.15
*P*-value		0.34	0.04
All			
*N*	200	200	110
MCC	0.50	0.51	0.47
Difference		0.00	0.03
*P*-value		0.78	0.04

Each data set was divided into single-chain and multi-chain categories. For each category, a two-tailed paired *t*-test was performed to test the statistical significance of difference between DEPTH MCC values and those of ConCavity and MetaPocket 2.0. *P*-values from two-tailed paired *t*-test are reported. N denotes the size of the dataset over which the comparisons were made.

By incorporating evolutionary information, the enhanced DEPTH ligand-binding site prediction improves over our previous method (MCC: 0.39) (7). A statistical analysis of DEPTH with ConCavity and MetaPocket 2.0 was performed (Table 3). Although DEPTH (0.63) is not as sensitive as ConCavity (0.80) and MetaPocket 2.0 (0.71), its predictions are more specific (DEPTH specificity: 0.92, ConCavity specificity: 0.87, MetaPocket 2.0 specificity: 0.89) and more precise (DEPTH precision: 0.48, ConCavity precision: 0.43, MetaPocket precision: 0.43).

**Table 3.** Statistical analysis of binding residues predictions of DEPTH, ConCavity and MetaPocket 2.0

Methods	N	TP	FP	TN	FN	Sensitivity	Specificity	Accuracy	Precision
DEPTH	200	0.07	0.07	0.82	0.04	0.63	0.92	0.89	0.49
ConCavity	200	0.08	0.11	0.78	0.02	0.80	0.87	0.87	0.43
MetaPocket 2.0	110	0.08	0.10	0.79	0.03	0.71	0.89	0.87	0.43

TP, FP, TN, FN represent the mean values of true positive, false positive, true negative and false negative rates over the testing set, respectively.

The testing set of 200 protein structures (for MetaPocket 2.0 comparisons, the size of the dataset was 110) consists of 12 020 binding site and 112 035 non-binding site residues. The average chain lengths of single- and multi- chain protein are 308 and 277, respectively. Of 120 multi-chain proteins, 77 are dimers, 3 are trimers, 22 are tetramers and the remaining 13 consist of five or more chains.

The predictions of DEPTH and ConCavity partially overlap with each other. Of all predictions made by DEPTH, 67.6% overlap with ConCavity. Of the consensus predictions by the two methods, 61.3% are true binding sites. In all, 49.4% of binding sites were predicted by both methods, and 86.3% of all binding sites were predicted by at least one of the methods (see Supplementary Figure SB1).

### Ionizable amino acid pK_a_ prediction

Using the known pK_a_ values of 367 residues in the training set, the coefficients of the linear combination of environment features [Equation (7)] were optimized (Table 4). Using these optimized values, pK_a_ predictions were made on 60 residues in the testing set (Supplementary Table SC1). On average, the root mean squared deviations (RMSDs) of our predictions were ∼0.96 pH units away from that of the experimentally determined values. Our predictions for ASP were closest to the experimentally determined values (RMSD = 0.71), whereas predictions for HIS were the farthest (RMSD = 1.26).

**Table 4.** RMSD of predicted pK_a_ from experimentally determined values, in pH units

Residue type	model pK_a_ (pH units)	c_0_	c_1_	c_2_	c_3_	c_4_	c_5_	RMSD (pH units)	
								Training set (size)	Testing set (size)
ASP	3.8	−2.18	0.29	0.47	−0.61	0.16	−0.15	1.02 (112)	0.71 (15)
GLU	4.5	−1.91	−0.1	0.79	−0.19	0.26	−0.09	0.83 (125)	1.07 (15)
HIS	6.5	3.13	−0.04	−0.54	0.28	−1.12	−0.83	1.14 (60)	1.26 (15)
LYS	10.5	4.22	−0.21	−0.19	−0.01	−7.65	−1.81	0.86 (70)	0.80 (15)
Total								0.94 (367)	0.96 (60)

c_0_–c_5_ are the coefficients of the linear recombination [Equation (7)].

We compared our predictions with those made by other methods including (i) Molecular dynamics/generalized-Born/thermodynamic integration (MD/GB/TI), with and without water (46), (ii) PROPKA (27), (iii) Geometry-dependent dielectric method (47), (iv) microenvironment screened Coulomb potentials (Microenv SCP) (48), (v) EGAD (49), (vi) Monte Carlo sampling with continuum electrostatics (MCCE) (50) and a Quantum mechanics/molecular mechanics (QM/MM) method (51) (Supplementary Table S5). The values of the testing set pK_a_s predicted from the methods listed earlier in the text, except PROPKA were obtained from literature (52). PROPKA 3.0 was run over the web server (http://propka.ki.ku.dk/) using default parameters.

In terms of the error in predicting pK_a_s, our predictions were significantly better (at 95% confidence using a Wilcoxon paired sign rank test) than the predictions of MD/GB/TI, Geometry-dependent dielectric method and EGAD (Table 5). Our results were on par with the PROPKA 3.0 and MCCE methods. Only QM/MM (0.30 pH units over five predictions) and Microenv SCP (0.70 pH units over 43 predictions) methods have lower pK_a_ errors than our predictions (0.96 pH units). Though the Microenv SCP method is statistically superior to our simple empirical method, we are closer to the experimentally determined value in 18 and worse in only 21 of the 43 common predictions.

**Table 5.** RMSDs of pK_a_ prediction of DEPTH and other methods to experimentally determined values

	MD/GB/TI with waters	MD/GB/TI without waters	PROPKA3.0	Geom dep dielectric	Microenv SCP	EGAD	MCCE	QM/MM	DEPTH
ASP	1.9 (4)	1.3 (15)	0.7 (15)	0.8 (14)	0.8 (12)	0.8 (10)	1.4 (12)	0.3 (1)	0.7 (15)
GLU	1.9 (3)	1.1 (15)	1.0 (15)	0.9 (14)	0.7 (13)	1.2 (8)	0.9 (14)	0.3 (4)	1.1 (15)
HIS	1.7 (7)	1.9 (15)	1.6 (15)	1.3 (15)	0.5 (9)	1.4 (7)	1.6 (9)		1.3 (15)
LYS	2.5 (1)	0.9 (15)	0.7 (15)	0.8 (9)	0.6 (9)		1.1 (11)		0.8 (15)
Total	1.9 (15)	1.4 (60)	1.1 (60)	1.0 (52)	0.7 (43)	1.2 (25)	1.3 (46)	0.3 (5)	1.0 (60)
*P*-value	<0.001*	<0.0001*	0.48	0.01*	0.02*	0.04*	0.45		

The number of predictions are given in parentheses. The *P*-values listed are from a Wilcoxon paired sign rank test comparing the DEPTH to the other methods.

*Indicates that statistically significant difference.

### Server description

Our server computes depth at the atomic/residue level and as applications, calculates cavity volumes, predicts the location of small molecule-binding sites and predicts the pK_a_ of ionizable amino acid residues. The web server is freely accessible without login requirements at http://mspc.bii.a-star.edu.sg/depth. Users have a choice of uploading a protein structure (in PDB format) or specifying the four-letter PDB code. The optimal values of parameters are set by default on the server. Users have the option to override the default values to cater to specific biological systems. Help pages provide information about the program and its different parameters.

The results of the computation/prediction are returned in pictorial representation and/or rendered using the Jmol viewer (http://www.jmol.org/), with appropriate accompanying figure legends. Users can download the results in tab-delimited and/or PDB formats. All results will be stored for up to 30 days. Stand-alone versions of the programs to compute depth, ASA and predict binding site residues are all available for download.

## DISCUSSION

The new version of our server re-establishes the importance of depth as a measure of determining several physical features of proteins. Having previously established its general utility, we have added three new application features—computing the sizes of cavity volumes, predicting ligand-binding sites and predicting pK_a_s of ASP, GLU, LYS and HIS residues.

Computing the depth of amino acid residues in proteins forms the basis of estimating cavity sizes within proteins. Although computing depth, water molecules from a solvating box are divided into bulk and non-bulk waters. The non-bulk waters usually occupy cavities. A Voronoi method was used to estimate the volumes of these cavities. Our volume estimates scale linearly with the experimentally measured change in free energy associated with cavity creating mutations, making it an accurate predictor of protein stability on mutation. Our relatively swift running program could also be used to help design cavity-filling mutations to structurally stabilize proteins.

Some of the cavities have the capability of binding small molecule ligands. We have refurbished our earlier method to predict such sites by adding evolutionary information in addition to the depth-related predictions of plausible binding site residues. Our method produces results that are now on par with the best prediction programs. Though the identification of binding sites is based on a relatively coarse measurement involving residue depth, our predictions are more specific and precise when compared with the predictions made by ConCavity and MetaPocket 2.0. DEPTH is an attractive and simple tool for functional annotation as well as finding suitable drug-docking sites.

Depth is a simple yet informative measure of protein internal environment. Several physical properties of proteins correlate well with depth. In this study, we have showcased this utility of depth in helping predict the pK_a_ of ionizable amino acid residues. Benchmarking results show that our empirical method is statistically indistinguishable from other methods such as MCCE and PROPKA 3.0, while being superior to the methods MD/GB/TI, Geometric dependent dielectric and EGAD. Although our predictions of the pK_a_’s of Histidine in general are a little weak, the method is rapid and performs at almost the same level of accuracy than other more sophisticated methods.

The DEPTH server is simple to use, and the output is presented to the users either visually on the output page or links are provided for downloading results. Given the proven general utility of depth and its correlation to several physical features, we hope to grow this server by adding more biologically relevant applications. Depth should be included as a standard measure in structural studies related to proteins and their functions.

## AVAILABILITY

The server is freely accessible at http://mspc.bii.a-star.edu.sg/depth/ and Supplementary Data are available at http://mspc.bii.a-star.edu.sg/tankp/benchmark.html.

## SUPPLEMENTARY DATA

Supplementary Data are available at NAR Online: Supplementary Tables A1–C3 and Supplementary Figure B1.

## FUNDING

Funding for open access charge: Biomedical Research Council (A*STAR), Singapore.

*Conflict of interest statement*. None declared.
